# Structural Model of the hUbA1-UbcH10 Quaternary Complex: *In Silico* and Experimental Analysis of the Protein-Protein Interactions between E1, E2 and Ubiquitin

**DOI:** 10.1371/journal.pone.0112082

**Published:** 2014-11-06

**Authors:** Stefania Correale, Ivan de Paola, Carmine Marco Morgillo, Antonella Federico, Laura Zaccaro, Pierlorenzo Pallante, Aldo Galeone, Alfredo Fusco, Emilia Pedone, F. Javier Luque, Bruno Catalanotti

**Affiliations:** 1 Kedrion S.p.A., Sant 'Antimo (Na), Italy; 2 Istituto di Biostrutture e Bioimmagini, Consiglio Nazionale delle Ricerche, Napoli, Italy; 3 Istituto di Endocrinologia ed Oncologia Sperimentale Consiglio Nazionale delle Ricerche, Napoli, Italy; 4 Dipartimento di Farmacia, Università degli Studi di Napoli Federico II, Napoli, Italy; 5 Departament de Fisicoquímica and Institut de Biomedicina (IBUB), Facultat de Farmàcia, Universitat de Barcelona, Santa Coloma de Gramenet, Spain; National Institute for Medical Research, Medical Research Council, London, United Kingdom

## Abstract

UbcH10 is a component of the Ubiquitin Conjugation Enzymes (Ubc; E2) involved in the ubiquitination cascade controlling the cell cycle progression, whereby ubiquitin, activated by E1, is transferred through E2 to the target protein with the involvement of E3 enzymes. In this work we propose the first three dimensional model of the tetrameric complex formed by the human UbA1 (E1), two ubiquitin molecules and UbcH10 (E2), leading to the transthiolation reaction. The 3D model was built up by using an experimentally guided incremental docking strategy that combined homology modeling, protein-protein docking and refinement by means of molecular dynamics simulations. The structural features of the *in silico* model allowed us to identify the regions that mediate the recognition between the interacting proteins, revealing the active role of the ubiquitin crosslinked to E1 in the complex formation. Finally, the role of these regions involved in the E1–E2 binding was validated by designing short peptides that specifically interfere with the binding of UbcH10, thus supporting the reliability of the proposed model and representing valuable scaffolds for the design of peptidomimetic compounds that can bind selectively to Ubcs and inhibit the ubiquitylation process in pathological disorders.

## Introduction

UbcH10 is a member of the Ubiquitin Conjugation Enzymes, a component of the anaphase-promoting complex and a key regulator of cell cycle progression [1], as it induces the ubiquitination and degradation of cyclins A and B [2]. Previous studies have indicated that UbcH10 over-expression might be associated with the late stages of thyroid neoplastic transformation [3], and that high levels of UbcH10 correlate with most aggressive grade tumors in breast cancer [4]. Similar evidences have been found for several tumor types, such as ovarian [5], colorectal and brain cancers [6] and different lymphoma [7]. Moreover, in numerous cancer tissues the UbcH10 expression is relatively higher if compared with the adjacent nonmalignant tissues. All these evidences point out that the aberrant expression of UbcH10 could promote tumor expansion through dysfunction of mitotic progression, leading to deregulation of cell growth as confirmed in both thyroid [6] and breast carcinoma [8], where the interference with the UbcH10 expression significantly reduced the tumor cell proliferation. Therefore, UbcH10 appears to be a potential target for developing an anti-cancer therapy based on the suppression of its specific biological function.

**Figure 1:**
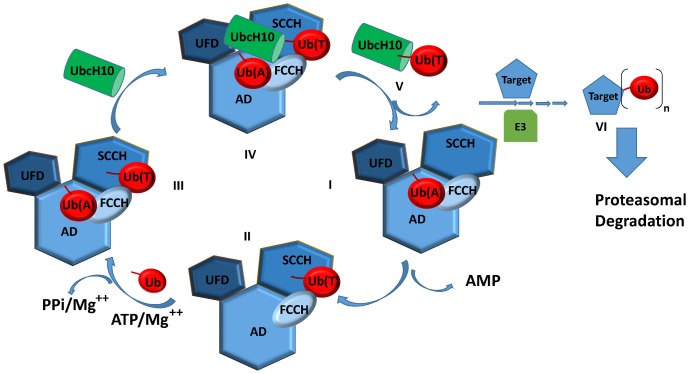
Ubiquitin conjugation cascade. UbA1 consists of four domains: the Adenylation domain (AD), the First Catalytic Cysteine Half-domain (FCCH), the Second Catalytic Cysteine Half-domain (SCCH) and the Ubiquitin Folding Domain (UFD). I) UbA1 catalyzes the adenylation of the Ub C-terminus in an ATP-dependent process in the AD domain; II) the activated Ub forms a thioester bond with a conserved catalytic cysteine in the SCCH domain of UbA1 [Ub(T)]; III) UbA1 is loaded with a second Ub molecule in the AD domain, followed by its C-terminal adenylation [Ub(A)]; IV) the ternary UbA1∼Ub(T)-Ub(A) thioester complex recruits E2 (e.g. UbcH10); V) the thioester-linked Ub is transferred to a conserved E2 cysteine (transthioesterification); VI) E3 mediates the binding of Ub to the target lysine ε-amino groups.

A key step in the discovery of inhibitors of the UbcH10-mediated ubiquitination is the comprehension of the structural and mechanistic features that mediate the conjugation of proteins to ubiquitin (Ub), a complex process that involves a three-step cascade mechanism characterized by growing specificity ([8]; see also ref. [9] for a recent review) (Figure 1). Thus, the Ubiquitin-Activating Enzyme (UbA1, also known as E1) initiates the ubiquitination cascade by catalyzing the ATP-dependent adenylation of the Ub C-terminus (step I). The high-energy anhydride bond thus formed is attacked by the E1 active site cysteine (C632 in human UbA1), forming a thioester bond between E1 and Ub (step II). Then, Ub is transferred to the active site cysteine of an Ub-Conjugation Enzyme (denoted E2), a process promoted by the non-covalent binding of a second Ub molecule in the adenylation site (steps III and IV). Finally, Ub is conjugated to its substrate with the aid of a protein ligase (known as E3), resulting in the covalent linkage of the Ub C-terminus to the ε-amino group of a lysine in the substrate (steps V and VI). In humans, there are two E1 enzymes (UbA1 and UbA6) [10], over 30 distinct forms of E2 and about 500–1000 forms of E3, which is largely responsible for conferring specificity to ubiquitylation [11].

The preceding mechanism is common to the Ubiquitin-like proteins (Ubl), a class of signaling proteins involved in cellular homoeostasis [12]. A number of X-ray and NMR studies (reviewed in [12]–[14]) have examined the structural features of the recognition between Ub and Ubl (SUMO and NEDD8) with E1, while only few studies were focused on the E1–E2 interaction, including the complex between APPBP1-Uba3∼NEDD8/NEDD8/MgATP/Ubc12 [13], and the construct obtained by crosslinking the catalytic cysteines of the UbA1∼Ubc4/MgATP [14]. While they reveal a general preservation of the E1 structure, they have disclosed the existence of significant structural differences, particularly in the SCCH (Second Catalytic Cysteine Half-domain) and UFD (Ubiquitin Folding Domain) regions, which highlight the intrinsic flexibility of E1 for accommodating both Ub and E2. However, to the best of our knowledge, there is not a complete 3D model of the quaternary complex required for the transfer of Ub to the E2 Ubiquitin Conjugation Enzyme.

In this paper we describe a computational and experimental strategy to build up the first structural model of the transient tetrameric complex between the doubly Ub-loaded human UbA1 (hereafter denoted UbA1∼Ub(T)-Ub(A)), and UbcH10, as a member of the E2 family. By combining homology modeling, protein-protein docking and molecular dynamics (MD) simulations, the structural features of the proposed model have allowed us to identify the regions that mediate the recognition between the interacting proteins. In turn, this information has been used to examine the reliability of the structural model through experimental assays performed to evaluate the binding affinities of a number of short peptides that were suitably chosen from the contact regions between interacting partners in the complex. Overall, this information can be valuable to gain insight into the specificity of the recognition between E1 and E2 partners, as well as for the design of peptidomimetic compounds that can bind selectively to E2s and inhibit the ubiquitylation process in pathological disorders.

## Materials and Methods

### Homology building

The amino acid sequence of human UbA1 (hUbA1) was retrieved from the National Center for Biotechnology Information (http://www.ncbi.nlm.nih.gov; accession ID P22314). To find suitable templates for homology modelling, a BLASTP [15] search was performed against the Protein Data Bank (PDB) [16]. At the beginning of this work, the search identified three templates: i) the crystal structure of mouse Ubiquitin-Activating Enzyme (PDB code 1Z7L; 2.8 Å resolution) [17], which covers 25% of the query sequence corresponding to the SCCH domain (sequence identity of 96%), ii) the crystal structure of the *Saccharomyces cerevisiae* UbA1 (scUbA1) - Ub complex (PDB code 3CMM; 2.7 Å resolution) [18], which covers 98% of the query sequence (sequence identity of 53%; similarity 71%), and iii) the NMR solution structure of a fragment of mouse UbA1 (PDB code 2V31) [19], which covers 10% of the query sequence corresponding to the FCCH region, with sequence identity of 93%. This latter structure showed that only the core region of FCCH was structured. Therefore, homology building was accomplished by using 1Z7L as template for the hUbA1 SCCH region (residues 629–884; hUbA1 numbering will be followed unless otherwise noted), and 3CMM as template for the AD, FCCH and UFD domains (residues 1–628 and 885–1057). Finally, since chains A and C in the X-ray structure 3CMM differ by a rigid-body rotation of the UFD domain, hUbA1 was modeled using the two monomers, leading then to two models hereafter designated UbA1_A and UbA1_C.

The ClustalW2 (http://www.ebi.ac.uk/Tools/msa/clustalw2/) [20] program was used for sequence alignment. The 3D structure of the target protein was modeled using SWISSMODEL [21]. The secondary structure of the target protein was assigned using DSSP [22]. Coordinates for two loops with undetermined coordinates in the UbA1 template structure (residues 812–824 and 964–969) were built up using the loop building ProMod tool [23] by scanning through the loop database in SWISSMODEL. The models were refined on the basis of energy minimization by GROMOS96 [24] and the models were validated for the 3D–1D profile with VERIFY3D [25], non-bonded interactions with ERRAT2 [26] and stereochemical qualities with PROCHECK [27] and WHATCHECK [28]. The comparison of the final model with the recently released structure of *Schizosaccharomyces Pombe* UbA1 (spUbA1; PDB code 4II3) revealed similar homology parameters with hUbA1 (covered sequence 94%; sequence identity of 54%; similarity 70%) and a RMSD for the backbone atoms of 1.6 Å, thus confirming the reliability of the model.

### General strategy for docking calculations

The 3D model of the quaternary complex between hUbA1, two Ub molecules and UbcH10 was determined by using an experimentally-guided incremental strategy that relies on the building and refinement of models for the dimeric and trimeric complexes. Thus, we first explored the recognition between hUbA1 and Ub, leading to the UbA1∼Ub(T) complex (Ub(T) stands for Ub bound to E1 through a thioester bond). Next, this model was used to build up the ternary UbA1∼Ub(T)-Ub(A) system (Ub(A) denotes Ub bound to the AD domain). Finally, this model was the starting point for assembling the quaternary complex, UbA1∼Ub(T)-Ub(A)/UbcH10. To this end, we adopted a computational approach that combines protein-protein docking, guided by the available structural information, and subsequent refinement through MD simulations (see below).

In order to generate the structural models, two docking programs were used: HADDOCK [29] and RosettaDock [30]. HADDOCK uses experimentally derived data, in conjunction with the available structures, to carry out flexible data-driven docking of proteins. Residues that are known to be implicated in the protein-protein recognition are designated active and are used to introduce suitable restraints to drive the docking process (i.e, the so called ambiguous interactions restraints; AIRs). HADDOCK expert interface was used to generate a reasonable rough complex, which was subsequently refined with the HADDOCK refinement interface.

To assess the initial orientation of the interacting partners in order to check the suitability of the restraints to be imposed in HADDOCK calculations (i.e., the extension and solvent accessibility of the region comprising passive residues, which are solvent-exposed residues that surround the active ones) the mutual complementarity of the interacting partners was first explored by superposing the structures of Uba1 and Ub(T) in the X-ray structure of the APPBP1-Uba3∼NEDD8-NEDD8-Ubc12 complex [13] (PDB entry 2NVU). A list of the restraints used in calculations is given in Supporting Information (Table S1).

Finally, the RosettaDock server performs a local docking searching for conformations near the starting 3D structure in order to find the optimal fit between the partners. It was then used to calibrate the models derived from HADDOCK.

### The UbA1∼Ub(T)-Ub(A)-UbcH10 complex

Following the incremental docking strategy, the dimeric UbA1∼Ub(T) system was generated using as input structures the previously generated hUbA1_A and hUbA1_C models, and the NMR structure of human Ub (PDB ID 2K6D) [32]. For HADDOCK calculations, the active residues were only those involved in covalent interactions, i.e. UbA1 Cys632 and Ub Gly76, and passive residues were defined as neighboring residues in a range of 8.5 Å from the active ones. Residues in the Ub tail (residues 70–76) and in the loop above the catalytic cysteine, whose coordinates were undetermined in template structures (residues 803–819), were set as fully flexible during all stages of the docking protocol. Since RosettaDock accepts a maximum of 600 residues, docking was performed using a truncated form of UbA1 that retains the residues pertaining to the interaction domain. Taking into account that RosettaDock allows the sliding of proteins around 8 Å, a binding region that includes residues 216–296 and 627–888 was defined.

The ternary complex was generated taking into account experimental information taken from the PDB structure 3CMM, in which Ub is bound to the AD domain of scUbA1. In HADDOCK calculations the active residues were those known to participate in the binding between UbA1 (Arg239, Asp576, Tyr600) and Ub (Asp32, Arg72, Gly75, Gly76). Passive residues were automatically defined as neighbors in a range of 8.5 Å from active residues. Besides the Ub tail, full flexibility was also given to residues of the UbA1 crossover loop (residues 592–630) to facilitate the accommodation of the Ub tail.

Finally, to build up the 3D model of the quaternary complex, the UbcH10 structure was taken from PDB ID 1I7K. Let us note that this structure is functionally active even though it lacks the first 30 residues at the N-terminus [33]. Note also that Ser114 in the crystal structure was mutated to Cys to restore the native sequence. In order to enhance the sampling in predicting the quaternary complex, four starting structures of the UbA1∼Ub(T)-Ub(A) complex were generated by combining the two UbA1 models (UbA1_A and UbA1_C) with two orientations of Ub in the UbA1∼Ub(T)complex (denoted Ha and Hb; see below). Hence, a total of four ternary models were used to build up the 3D structure of the quaternary complex. Active residues in HADDOCK calculations comprised those involved in E1–E2 interactions on the basis of mutagenesis studies [13], [31], [41]–[43]: Glu1037, Asp1047 and Glu1049 in UbA1 (numbering for the UbA1-Ub2 complex), and Lys33′ and Gln37′ in UbcH10. Moreover, the two catalytic cysteine residues (Cys632 in UbA1 and Cys114′ for UbcH10) involved in the transthiolation process were also treated as active residues in order to guide the complex formation. Passive residues were defined as neighbors in a range of 8.5 Å from active residues. Residues of the UbA1 crossover loop (residues 592–630) and the Ub tail (residues 70–76) were also flexible. Each ternary model was docked twice with UbcH10 structure yielding a total of 80 clusters.

### Molecular Dynamics

MD simulations were performed to refine the different complexes. To this end, each complex was immersed in a pre-equilibrated octahedral box of TIP3P water molecules, and the system was neutralized. The final systems contained between 93000 and 99000 atoms. All simulations were performed with the parmm99SB force field [34]. The thioester bond between Ub(T) Gly76 and UbA1 Cys362 was manually added, and suitable force field parameters were derived using CH_3_CH_2_SCOCH_3_ as a representative model. The AMP position was derived from the ATP molecule as found in the PDB structure 2NVU. To this end, the AD domain of the UbA1∼Ub(T)-Ub(A) model was superimposed to the AD domain of NAE1/UbA3. On the other hand, the phosphodiester bond between Ub(A) Gly76 and AMP was manually added, and the force field parameters for the phosphodiester linkage between UbA1 Cys632 and Ub(T) Gly76 were derived using CH_3_OP(O)_2_OCOCH_3_ as a model system.

For each complex the geometry was minimized in four steps, which involve: i) water molecules and counterions (3000 steps of steepest descent and 7000 steps of conjugate gradient), ii) hydrogen atoms in the protein (500 steps of steepest descent and 4500 steps of conjugate gradient), iii) then, hydrogen atoms, water molecules and counterions (3500 steps of steepest descent and 11500 steps of conjugate gradient, and iv) finally the whole system (2500 steps of steepest descent and 8500 steps of conjugate gradient). Thermalization of the system was performed in four steps of 60 ps, increasing the temperature from 50 to 298 K. Concomitantly, the atoms that define the protein backbone were restrained during thermalization using a variable restraining force. Thus, a force constant of 20 kcal^.^mol^−1^ Å^−2^ was used in the first stage of the thermalization and was subsequently decreased by increments of 5 kcal^.^mol^−1^ Å^−2^ in the next stages. Then, an additional step of 250 ps was performed in order to equilibrate the system density at constant pressure (1 bar) and temperature (298 K). Finally, an extended trajectory was run using a time step of 2 fs. SHAKE was used for those bonds containing hydrogen atoms in conjunction with periodic boundary conditions at constant pressure and temperature, particle mesh Ewald for the treatment of long range electrostatic interactions, and a cutoff of 10 Å for nonbonded interactions. The structural analysis was performed using in-house software and standard codes of Amber 12.

### Steered Molecular Dynamics and refinement of the final complex

Comparison of the final MD structures and the recently solved X-ray structure of Uba1 in complex with Ubc4 (PDB entry 4II2; [14]) showed that the loop masking the hUbA1 catalytic cysteine (Cys-cap loop) prevented a close packing between UbcH10 and the ternary complex. Accordingly, the protein-protein interface was refined by means of steered molecular dynamics (SMD) simulations, which were set up using Amber 12. To this end, the Cys-cap loop (residues 801–825) was deleted and capping groups were added to the newly formed terminals. The distance between the sulfur atom of the UbcH10 catalytic cysteine (C114) and the carbon atom of the terminal carboxy group of Ub(T) was constrained to 3 Å in 4 steps: i) from the initial distance (9.4 Å) to 7 Å in 0.5 ns with a force constant of 5 kcal/mol; ii) from 7 to 4 Å in 1.5 ns with a force constant of 5 kcal/mol; iii) from 4 to 3 Å in 2 ns with a force constant of 10 kcal/mol; iv) and finally from 3 to 2.5 Å in 4 ns with a force constant of 20 kcal/mol. At the end, the system was rebuilt by adding the removed Cys-cap loop (UbA1 residues 801–825), equilibrated with suitable constraints in order to relax the residues in the Cys-cap loop, and finally subjected to an unrestrained MD (50 ns) simulation.

### Binding free energy evaluation and virtual alanine scanning

Binding free energies of the docking solutions and sampled in MD simulations were estimated using the Solvated Interaction Energy (SIE) method [35], as implemented in the Sietraj program [36]. Analysis of the MD trajectory was carried out by calculating SIE on a 0.1 ns interval at the end of the trajectory. On the other hand, the contribution of specific residues to the binding between interacting proteins was examined by using alanine scanning [37], [38].

### GST-UbcH10 preparation for experimental binding assays

The PCR product was cloned into pGEX-4T1 expression vector (GE Healthcare), leading to a protein with a cleavable N-terminal GST tag (GST-UbcH10). *E. coli* BL21 (DE3) RP strain was transformed with the recombinant plasmid for GST-UbcH10. Overnight cultures were used to inoculate 500 ml LB medium containing 50 µg/ml ampicillin, and protein induction was performed by the addition of 1 mM IPTG at 22°C when an OD_600_ value of 0.7 was reached. After approximately 16 h the cells were harvested and the proteins were isolated by sonicating cell pellets resuspended in 30 ml PBS1X in the presence of an EDTA free protease inhibitor cocktail (Roche Diagnostics). The crude cell extract was cleared by centrifugation at 18000 rpm and the supernatant was loaded onto a 1 ml GST-trap column connected to AKTA FPLC system (GE-Healthcare) equilibrated with binding buffer PBS1X. After washing with ten volumes of binding buffer, a single elution step was performed with 50 mM TrisHCl, 10 mM reduced glutathione. The fractions containing GST-UbcH10 were pooled and extensively dialyzed against PBS1X at 4°C. The homogeneity of the protein was tested by SDS–PAGE and mass spectrometry.

### Peptides synthesis

A series of peptides chosen to mimic specific regions of the protein-protein interface (L1, L2, U1 U2, S1 and S2), as well as the L2- scrambled (ScrL2) and U1-scrambled (ScrU1) peptides were obtained by Fmoc solid-phase strategy. To mimic the fragment within the parent protein, the *N*- and *C*-terminus were acetylated and amidated, respectively. The syntheses were carried out with Novasyn TGR resin (substitution 0.25 mmol g^−1^). Coupling reactions were performed by using 10 equiv of Fmoc protected amino acids activated *in situ* with HBTU (9.8 equiv)/HOBt (9.8 equiv)/DIPEA (20 equiv) in DMF for 1 h. Fmoc protecting group was removed by treatment with 30% piperidine in DMF two times for 10 min. Before the cleavage from the resin, all peptides were acetylated or biotinylated at the *N*-terminus to obtain the corresponding derivatives. The acetylation reaction was carried out two times for 10 min using a solution of acetic anhydride (0.5 M)/DIPEA (0.15 M)/HOBt (0.125 M) in DMF. Biotinylated peptides were obtained using a solution of *N*-(+)-biotinyl-6-aminocaproic acid (2 equiv)/PyBop (2 equiv)/DIPEA (4 equiv) in DMF overnight. All peptides were cleaved off the resin by treatment with a mixture of TFA/H_2_O/ethanedithiol (EDT)/triisopropylsilane (TIS) (94:2.5:2.5:1v/v/v/v) for 3 h at room temperature. The resins were filtered and the crude peptides were precipitated with diethyl ether, dissolved in a H_2_O/CH_3_CN (1∶1 v/v) solution and lyophilized.

L1, L2, L2-scrambled, U2, S1 and S2 peptides were purified by preparative RP-HPLC on a Shimadzu system equipped with the UV-Vis detector SPD10A using a Phenomenex Jupiter Proteo column (21.2×250 mm; 4 µm; 90 Å) and a linear gradient of H_2_O (0.1% TFA)/CH_3_CN (0.1% TFA) from 5 to 70% of CH_3_CN (0.1% TFA) in 20 min at flow rate of 5 ml/min. U1 and U1-scrambled peptides were dissolved in H_2_O/CH_3_CN solution with TCEP to avoid S-S bridge formation and purified using a linear gradient of ammonium formate buffer 0.1 M (pH = 7.0) and ammonium formate buffer/CH_3_CN 0.1 M (pH = 7.0) (1∶1 v/v) from 20 to 65% of ammonium formate buffer/CH_3_CN 0.1 M (pH = 7.0) in 25 min at flow rate of 5 mL/min. The collected fractions containing the peptides were lyophilized. The identity and purity of peptides were assessed by an ESI-LC-MS instrument (ThermoFinnigan, NY, USA) equipped with a diode array detector combined with an electrospray ion source and ion trap mass analyzer using a Phenomenex Jupiter Proteo column (150×2 mm; 4 µm; 90 Å) and a linear gradient of H_2_O (0.1% TFA)/CH_3_CN (0.1% TFA) from 5 to 70% of CH_3_CN (0.1% TFA) in 15 min at flow rate of 200 µl/min for L1 and L2 peptides and from 20 to 80% of CH_3_CN (0.1% TFA) in 15 min for U1 peptides.

### ELISA assay

Wells were coated overnight at 4°C with 100 µg/ml GST-UbcH10 in PBS 1X, 1 mM TCEP, in the presence of an EDTA free protease inhibitor cocktail (Roche Diagnostics). Binding step was performed with different concentrations of biotinylated peptides L1, L2, ScrL2, U1, ScrU1, S2 (2.2, 11, 22, 44, 108 µM) in PBS 1X (with 1 mM TCEP for U1 and ScrU1). A blocking solution 1% BSA in PBS 1X, 0.05% Tween-20 was used. Washes were executed with PBS 1X, 0.05% Tween-20. To verify the interaction a 1∶10000 dilution of horseradish peroxidase-conjugated streptavidin (Sigma Aldrich) in 0.3% BSA, PBS 1X was incubated for 1 hour. The colorimetric reaction was carried out with SIGMA-FAST OPD reagent (Sigma Aldrich), according to the manufacturer's instructions. Finally, readings were performed at 490 nm on a Model680 MicroplateReader (Bio-Rad, Hercules, CA-USA), and data were recorded by Microplate Manager 5.2 program and elaborated by GraphPad Prism program. Negative control experiments with the fusion tag GST in coating were performed in the same conditions described above.

## Results and Discussion

In order to determine the 3D model of the tetrameric complex responsible for UbcH10 transthiolation and identify the regions involved in protein recognition, we have first built the trimeric complex formed by UbA1 with two Ubs, one covalently bound at UbA1 Cys632 (Ub(T)) through a thioester bond (indicated with the symbol ∼) and the other non-covalently bound at the AD site (Ub(A)) following an incremental docking procedure that follows the series of events leading to the quaternary system (Figure 1). The model of the quaternary complex was then experimentally validated by competitive binding assays using a series of peptides chosen for their contribution to the protein-protein interface in the 3D model (see below).

### Homology modeling of hUbA1

The structural model of hUbA1 was built up by using 1Z7L and 3CMM structures as templates for the hUbA1 SCCH region (1Z7L) and for the AD, FCCH and UFD domains (3CMM), respectively. Moreover, the two conformations of *S. cerevisiae* UbA1 (scUbA1) found in the X-ray structure 3CMM were considered, leading to 3D models named UbA1_A and UbA1_C (see Materials and Methods). The quality of the models was checked by considering a number of structural features, including stereochemical properties, the compatibility between the amino acid sequence and the environment of amino acid side chains, and the patterns of non-bonded interactions (see Table 1). The Ramachandran plots for the two UbA1 models showed that around 98% of the total residues were located within the allowed regions (88% in the most favored ones), and only 3 (UbA1_A) or 4 (UbA1_C) residues were found in disallowed regions (0.3%) (Figure S1). The global PROCHECK G-factor for UbA1_A and UbA1_C was −0.08 and −0.15, respectively, indicating that the two structures are acceptable, because the recommended value must be greater than −0.50. On the other hand, the VERIFY3D scores above the threshold of 0.2 (86.7% and 90% for UbA1_A and UbA1_C, respectively) also indicated good local structural environments. Finally, the ERRAT2 analysis, which examines the quality of non-bonded interactions, yielded an estimate above 95%, indicating that the two models exhibit interresidue contacts that compare well with the patterns observed in high-resolution structures.

**Table 1:** Structural models of hUbA1.

Protein	Procheck^1^				Errat2	Verify3D
	Most favoured	Allowed regions	Generously allowed	Disallowed	%	%(avg>0.2)
CMM_A	780 (88.1%)	98 (11.1%)	1 (0.1%)	6 (0.7%)	91.8	88.0
hUbA1_A	781 (87.9%)	93 (10.5%)	12 (1.3%)	3 (0.3%)	95.8	86.7
CMM_C	787 (88.3%)	90 (10.1%)	7 (0.8%)	7 (0.8%)	96.4	94.0
hUbA1_C	789 (88.6%)	95 (10.7%)	3 (0.3%)	4 (0.4%)	95.6	90.0

Validation results for the lowest energy models of hUbA1, compared with the corresponding templates.

1 Number of residues in the region (the percentage is given in brackets).

Taking into account the similar scores obtained for the two models and their structural resemblance (RMSD  = 1.2 Å), MD refinement was accomplished only for UbA1_C. A stable structure was obtained after the first 5 ns of the trajectory (Figure S2; A). The increase in the RMSD was mainly due to structural rearrangements of the domains present in UbA1, leading to an average displacement of *ca*. 6 Å. Nevertheless, the structure of each domain was very stable along the trajectory, as demonstrated by the stability of the RMSD of the single domains (Figure S2; B).

### hUbA1∼Ub(T) and hUbA1∼Ub(T)-Ub(A) complexes

**Figure 2:**
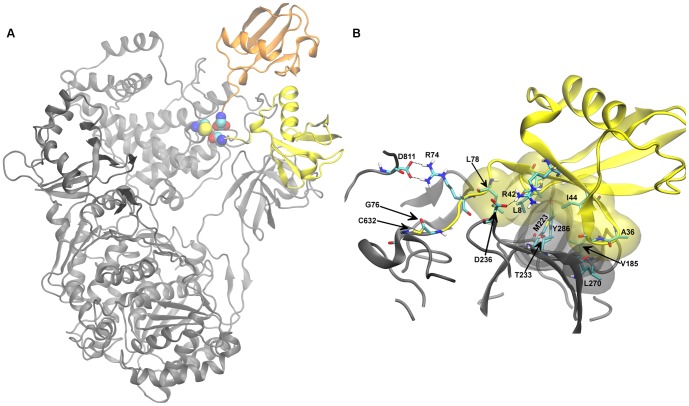
Model of hUbA1-Ub(T) complex. A) Comparison of the best two binding modes of Ub resulting from HADDOCK calculation, Ha and Hb, shown in yellow and orange, respectively. Terminal Ub-Gly76 and catalytic UbA1-Cys632 are highlighted in spheres coloured by atom type. B) Detail of hUbA1∼Ub(T) interactions in the lowest energy MD frame (time 9.6 ns). Apolar hydrogens were omitted for sake of clarity. Colour code: hUbA1, grey; Ub(T), yellow; van der Waals interactions are highlighted with transparent Connolly surfaces. Carbons are in cyan, nitrogen in blue, oxygens in red, sulphur in yellow and hydrogens in white.

To build up the tetrameric complex between hUba1, Ub(T), Ub(A) and UbcH10, we first modeled the hUbA1∼Ub(T) thioester complex, which was subsequently used to dock a second Ub molecule in the AD domain. Modeling the binding mode of Ub(T) is challenged by the lack of structural and biochemical information about this interaction, and by the covalent linkage of Ub, which is an unusual feature in protein-protein docking. To this end, a multistep strategy that included the use of two protein-protein docking webservers, HADDOCK and RosettaDock, in order to disclose the non-covalent interfaces between the E1 and Ub(T), was adopted. Accordingly, we first docked Ub to hUba1 using HADDOCK by restraining the contact between Cys632 (UbA1) and Gly76 (Ub). Among the 9 clusters that embody the 200 best structures yielded by HADDOCK (Table S2), solutions were chosen on the basis of four criteria: i) the distance from the sulfur atom of Cys632 and the carboxylic oxygen of Gly76, ii) the total score, iii) the total number of poses, and iv) the buried surface area. The selected poses lead to a distance lower than 3.8 Å, and are characterized by a high score, a large number of poses, and a large burial of surface area (see Table S2). These poses (denoted Ha and Hb) mainly differ in the orientation of Ub relative to the SCCH domain (Figure 2-A). In the lowest energy solution (Ha), Ub forms contacts with SCCH, mainly through ionic and polar interactions, and FCCH, primarily through hydrophobic interactions via the Ile44 patch, which is known to be involved in other non-covalent interactions of Ub, such as in the recognition of UbcH5c, UEV and GLUE domains [39]. In the second pose (Hb), Ub only showed polar contacts between residues in the Ub tail with the SCCH domain.

The two poses were then checked using RosettaDock. The best ranked solution turned out to be very similar to the best HADDOCK solution (Ha), as noted in a RMSD of 0.82 Å. In contrast, calculations started from pose Hb yielded solutions that showed significant structural differences with regard to the initial structure. Therefore, due to the structural consistency of pose Ha, it was chosen as a model of the hUbA1∼Ub(T) complex and subsequently refined by MD simulations, which led to a stable trajectory after the first 2 ns (see Figure S2-A, C). The refined structure supports the hydrophobic contacts between residues Leu8, Ile44, Val70 and Leu73, which interact with FCCH residues Y286, Met223, Val277, Leu178 and Thr233. The hydrophobic interactions involving the Ile44 patch were also reinforced by ionic interactions between Arg42 (Ub(T)) and Asp236 and between Arg74 (Ub(T)) and Asp811 (Figure 2-B).

**Figure 3:**
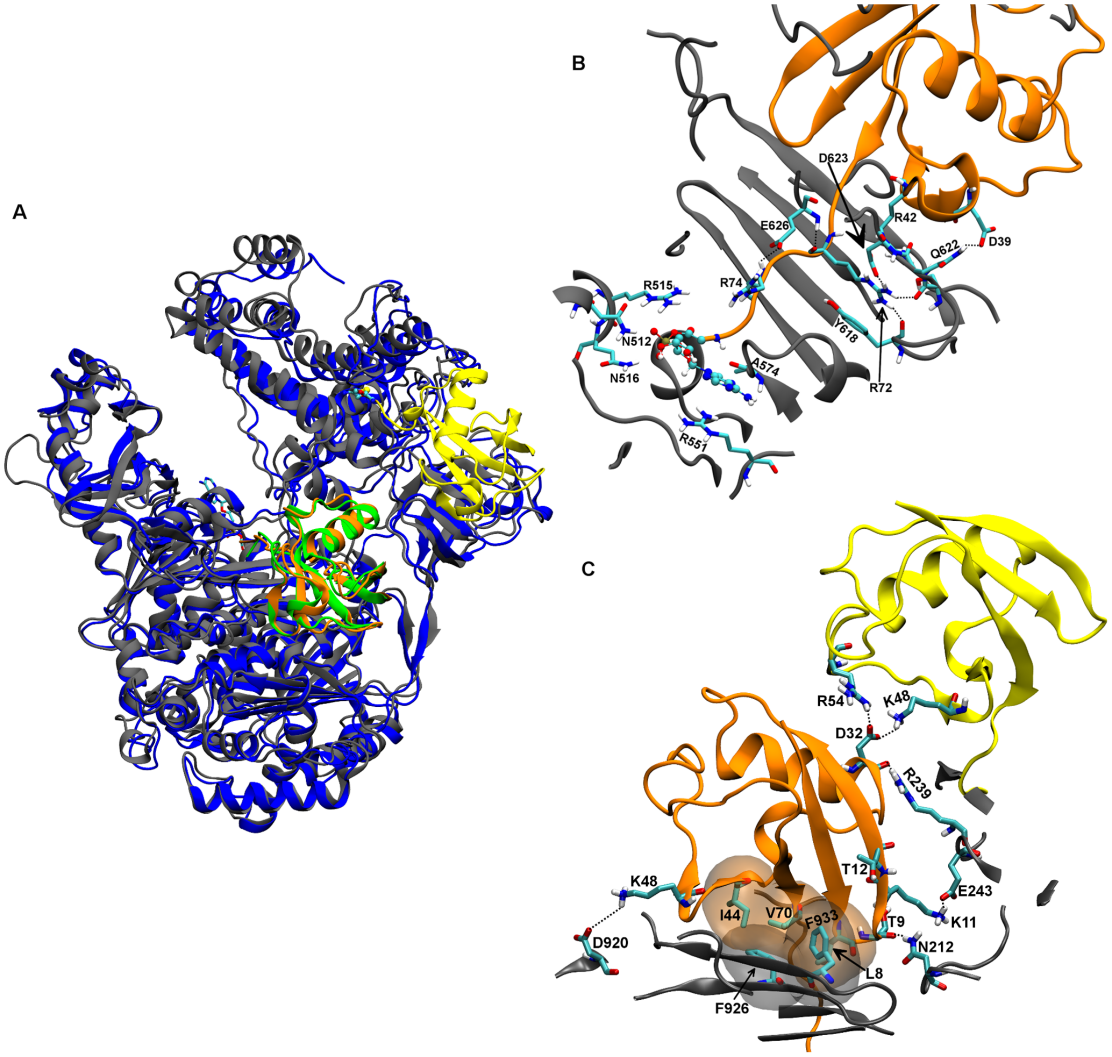
Model of hUbA1-Ub(T)-Ub(A) complex. A) Superimposition of the hUbA1∼Ub(T)-Ub(A) model C_Ha on the crystal structure 3CMM_C; B) Detail of the main interactions of Ub(A) and AMP in the hUbA1 loop pocket; C) Detail of the main interactions between Ub(A) and hUbA1: hydrophobic and polar interface. AMP is highlighted in CPK. Apolar hydrogens were omitted for sake of clarity. Colour code: hUbA1, grey; Ub(T) yellow; Ub(A), orange; scUbA1, blue; scUb(A), green.

The trimeric complex was obtained through docking of Ub to the AD site and subsequent MD refinement, which led to a stable trajectory after the first 2 ns (see Figure S2-A, D). The 3D structures closely resembled the X-ray template 3CMM (RMSD of 1.1 Å; Figure 3A). Three different interfaces might be identified: i) the loop pocket defined by hUbA1 residues Tyr618, Ser621, Glu626, Arg515, Asn512, Asn516 and Arg551 interacting with Ub(A) tail residues Arg72, Arg42 and Arg74 and AMP (Figure 3, B); ii) an hydrophobic patch formed by the Ub residues Leu8, Ile44 and Val70 that form contacts with an hydrophobic area in the hUbA1 AD region formed by residues Phe933 and Phe926 (Figure 3-C); and iii) the polar interface formed by Ub(T) residues Thr9, Lys11, Thr12, Asp3 interacting with the FCCH region, mainly with residues Glu243, Arg239 and Asn212 (Figure 3-C). Moreover, interactions between Ub(T)-Lys48 and Asp920 and Glu938, not present in the 3CMM structure, were also found.

Finally, during the submission of the article, a novel structure of the scUbA1 loaded with two ubiquitin molecules was released in the PBD with the name 4NNJ [40]. The superimposition of the model of hUbA1-Ub(T)-Ub(A) complex obtained with our incremental strategy to the crystal structure (chains C,D,E) leads to an rmsd of 1.5 Å (determined for the Cα carbon atoms), and showed a position of Ub(T) very similar to the pose C_Ha selected in our calculations (Figure S3), thus supporting the reliability of our model.

### hUbA1∼Ub(T)-Ub(A)-UbcH10 complex

The hUbA1∼Ub(T)-Ub(A) model was the starting point for the docking with UbcH10. In order to make a more exhaustive sampling, up to four different starting points were considered for docking calculations (see Materials and Methods). HADDOCK calculations yielded 80 clusters. The results were filtered by selecting only poses where the distance from the sulphur atom of the UbcH10 catalytic cysteine (Cys114) to the carbonyl carbon of the C-terminal Gly in Ub(T) was lower than 20 Å, considering this limit as indicative of the side of UbcH10 facing the SCCH region. Moreover, this criterion is consistent with the distance between the Cys residues involved in the transthiolation reaction in the crystal structure 2NVU, representing the tetrameric complex of the NEDD8 system. Only six clusters satisfied the distance cutoff. Among these clusters, Ub(T) adopted the Ha binding mode in five cases, and the Hb arrangement was found in a single case. This suggests that the Ha binding mode position was better suited to accommodate the E2 partner within the E1 groove with the catalytic cysteines facing each other. Table 2 shows the distinct poses ranked according to HADDOCK score as well as to the binding free energy of the complex calculated with the SIE method using the snapshots collected in the MD simulation. A_Ha_L2 emerges as the best pose according to HADDOCK score and SIE binding affinity. Both HADDOCK and SIE scores are consistent in suggesting C_Ha_R and C_Ha_L as feasible poses. The structures of these complexes differ by around 5.5 Å relative to A_Ha_L2. The A_Ha_L1 and A_Ha_R poses were structurally similar to A_Ha_L2 (rmsd of 3.3 Å) and Ca_Ha_R (rmsd of 4.3 Å), respectively. However, SIE calculations predict that they are less stabilized compared to A_Ha_L1 and A_Ha_R. Finally, C_Hb_R was rejected due to their low energetic score.

**Table 2:** Comparison of structural and energy data for selected docking results of the quaternary complex.

Docking	UbcH10 S-C114 HUbA1 S-C632	UbcH10 S-C114 Ub(T) C-ter G76	Haddock Score	SIE ΔG
A_Ha_L2	15,2	17,6	−147.7±11.2	−11.0
C_Ha_R	14,6	14,5	−126.7±5.4	−9,6
C_Ha_L	18,1	17,4	−125.0±5.4	−9,2
A_Ha_L1	16,9	15,1	−118.9±7.3	−8.0
A_Ha_R	11,3	11,8	−125.0±10.0	−7,6
C_Hb_R	16,7	18,5	−96.6±8.3	−3,6

The structural data include the distance (Å) from the sulphur of the UbcH10 Cys114 to the hUbA1 Cys632, and to the C-terminal Gly76 of Ub(T). The energy data report the score of the docked structures obtained from HADDOCK and from SIE (kcal/mol).

It is experimentally known that the N-terminal helix and β1β2 loop of E2 are directly involved in the formation of the complex [13], [31], [41]–[43]. In particular, mutational and structural studies disclosed the main role of two basic residues, conserved in the E2 family (positions 33 and 37 in UbcH10 numbering), in assisting the binding to E1. We have therefore examined the role played in the selected models by i) the conserved acidic residues of the UFD region of hUbA1 (i.e. Glu1037, Asp1047 and Glu1049) and ii) the conserved basic residues of the N-terminal helix of UbcH10 (i.e. Lys33′ and Gln37′). It is worth noting that while a basic residue in position 33 is conserved in all the members of the E2 family, position 37 shows a higher variation, albeit basic or polar residues are generally found in this position. To this end, the best three solutions (A_Ha_L2, C_Ha_R and C_Ha_L) were subjected to a virtual alanine scanning analysis in order to evaluate the contribution of these residues to the E1–E2 interaction. Even though the results (Table 3) did not show significant interactions (defined as ΔΔG ≥−0.5 kcal/mol) with Gln37′, the best three models showed a significant contribution to the binding of at least one residue from the N-terminal helix and one residue from the UFD region of UbA1. For the sake of comparison, no significant contribution was found for the mutations in the N-terminal helix for poses A_Ha_L1 and A_Ha_R. In fact, only a single mutation in hUbA1 (Asp1047→Ala) was found to lead to a significant destabilization. This finding, together with the structural resemblance to A_Ha_L2 and Ca_Ha_R and the lower SIE binding free energy (see above), led to their exclusion from further refinements.

**Table 3:** Alanine scanning.

Docking	ΔΔG hUbA1			ΔΔG UbcH10	
	E1037A	D1047A	E1049A	K33A	N37A
A_Ha_L2	0.0	−0.8	0.0	−0.8	−0.4
C_Ha_R	−0.2	−0.4	−1.5	−0.7	0.0
C_Ha_L	−0.3	−1.3	0.1	−1.0	−0.1
A_Ha_L1	0,0	−1,2	0,0	−0,2	−0,2
A_Ha_R	−0,2	−0,6	0,0	−0,3	0,1
C_Hb_R	−0,4	0,2	−0,6	−0,7	0,0

Results of the virtual alanine scanning (ΔΔG; kcal/mol) due to the mutation to Ala of residues Glu1037, Asp1047 and Glu1049 in hUbA1 and Lys33′ and Gln37′ in UbcH10 are reported.

**Figure 4:**
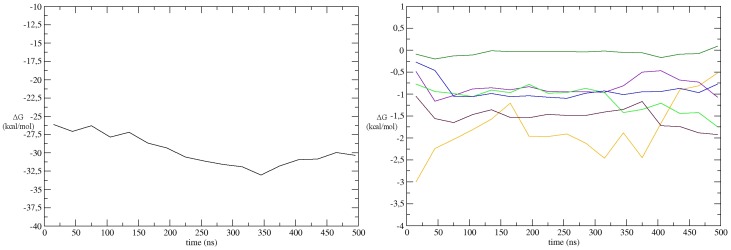
Energetic analysis. A) SIE values (kcal/mol) determined for the E1-E2 interaction along the trajectory (averaged for 20 ns windows). B) Contribution of key residues as derived from alanine scanning in UbcH10 N-terminal helix and the hUbA1 UFD region during the MD simulation of the model C_Ha_R. Colours: Glu1037, orange; Asp1047, violet; Glu1049, light green; Lys33′, bordeaux: Gln36′, blue; Gln37′, dark green.

**Figure 5:**
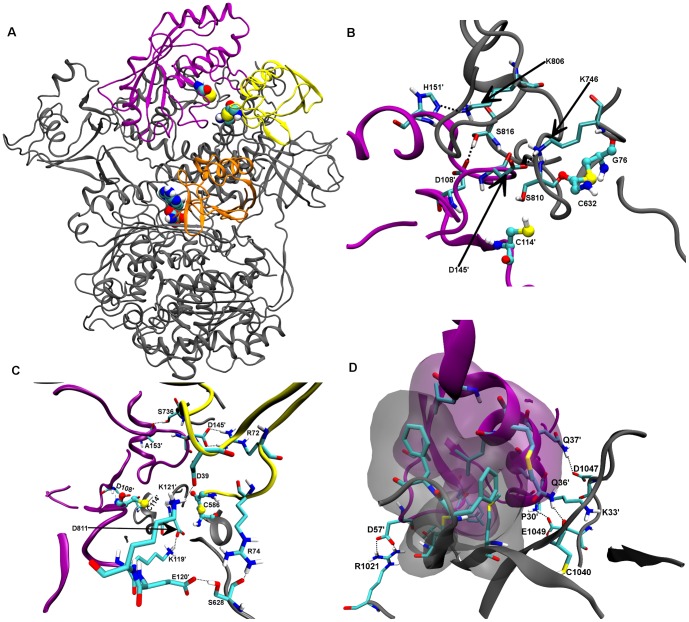
Model of the hUbA1-Ub(T)-Ub(A)-UbcH10 complex. A) Average structure of last 20 ns of MD simulation of the C_Ha_R model. The catalytic cysteines, the thioester bond and AMP were highlighted in spheres. Detail of interactions between B) UbcH10 and SCCH region, C) UbcH10 and Ub(T) and D) UbcH10 and UFD region. Catalytic cysteins and the thioester bond were highlighted in CPK. Apolar hydrogens were omitted for the sake of clarity. Colour code: hUbA1, grey; scUbA1, blue; Ub(T) yellow; Ub(A), orange; UbcH10, violet. The van der Waals interactions are highlighted with transparent Connolly surfaces.

The three models were further refined by running a series of 50 ns MD simulations, and the binding free energy was determined from SIE calculations performed for the snapshots sampled in the last four 10 ns windows. The results consistently showed that the best binding affinity was obtained for model C_Ha_R (−26.6±0.2 kcal/mol), it being more favorable by 6 and 9 kcal/mol compared to A_Ha_L (−20.2±1.4 kcal/mol) and C_Ha_L (−17.2±1.5 kcal/mol) models. On the basis of the preceding findings, the C_Ha_R model was further refined by extending the MD simulation to 500 ns. The analysis of the trajectory revealed a progressive stabilization of the complex, leading to a binding affinity close to −31 kcal/mol in the last 250 ns (Figure 4-A). The alanine scanning analysis also demonstrated that the residues known to be critical to E1–E2 complex formation contributed significantly to the protein-protein interaction with the only exception of Gln37′ (Figure 4-B). During the MD run we observed a change in hUbA1 associated to the rotation in opposite directions of the UFD and SCCH domains with respect to the AD domain (by 20° and of 13°, respectively, as calculated with DynDom [44]). This conformational change caused the widening of the groove defined by the three domains, thus allowing a closer contact between hUbA1 and UbcH10, leading to an increase of the interaction surface (Table S3) and the gradual decrease of the distance between the UbcH10 catalytic cysteine and the Ub(T) C-terminal glycine crosslinked to UbA1-Cys632 until it stabilised at around 8 Å (Figure S4). Analysis of the last 50 ns of the trajectory revealed the presence of two main interaction surfaces, which involve contacts between i) UbcH10 helix H1 and β1β2 loop and hUbA1 UFD domain, and ii) the hUbA1 Cys-cap loop and Ub(T) (Figure 5).

### Final refinement of the tetrameric complex

Although MD simulations led to a progressive refinement of the quaternary complex, the distance between residues Cys114 in UbcH10 and the terminal glycine of the crosslinked Ub(T) was still too large (∼8 Å; Figure S4) as to mimic a state that resembles the catalytic arrangement of the interacting proteins. Inspection of the final MD structure showed that a closer approach between hUbA1 and UbcH10 was prevented by the Cys-cap loop, which retained the orientation found in the PDB template 3CMM. In contrast, in the available structure of the E1–E2 complex (PDB structure 4II2) the Cys-cap loop is not assigned, thus suggesting a large flexibility in the covalent construct that mimics the thioester crosslinking event. We have therefore forced the approach of UbcH10 by using steering forces applied on the sulphur of the UbcH10 Cys114 toward the carbonyl group of the crosslinked Ub(T) C-terminal glycine, after manual removing of the Cys-cap loop. SMD simulations allowed us to reduce the distance between those atoms from 8 Å to 3.2 Å in 8 ns. After loop reconstruction, the final structure was refined in a 50 ns MD simulation, leading to a stable trajectory (Figure S5). This approach led to a closer fitting of UbcH10 into the groove defined by the UFD and SCCH domains, increasing the total interaction surface, especially between hUbcA1 SCCH and the UbcH10 region around the catalytic cysteine, in better agreement with the crystal structure of the E1–E2 crosslinked construct (Table S3). Moreover, SIE calculations revealed an increase of the binding energy to −42.7 kcal/mol.

Comparison of the refined model with the recently reported X-ray crystallographic structure of the trimeric complex of *S. pombe* Uba1-Ub-Ubc4 (PDB ID: 4II2) lends support to the theoretical 3D model of the quaternary complex. Thus, after deletion of the E2 partners (UbcH10 and Ubc4) and the additional Ub present in the quaternary complex, superposition of the backbone Cα carbon atoms leads to a positional rmsd of 2.5 Å, which indicates the similar structural arrangement of the AD, SCCH and UFD domains in the two complexes (see also Figure S6). Furthermore, retention of the E2 partners in the superposed structures leads to an rmsd value of 2.6 Å, thus suggesting a similar arrangement in the trimeric and quaternary complexes.

The analysis of the snapshots sampled in the last 20 ns of the trajectory allowed us to identify key interactions in the complex, which involve three interfaces: i) the contacts between the hUbA1 UFD domain and the UbcH10 helix H1 and β1β2 loop, ii) the interaction between the hUbA1 SCCH domain and Ub(T) with the region surrounding the UbcH10 Cys114', involving residues from the 3–10 helix and helices H2 and H3, and iii) the contacts between the hUbA1 crossing loop and Ub(A) with UbcH10.

**Figure 6:**
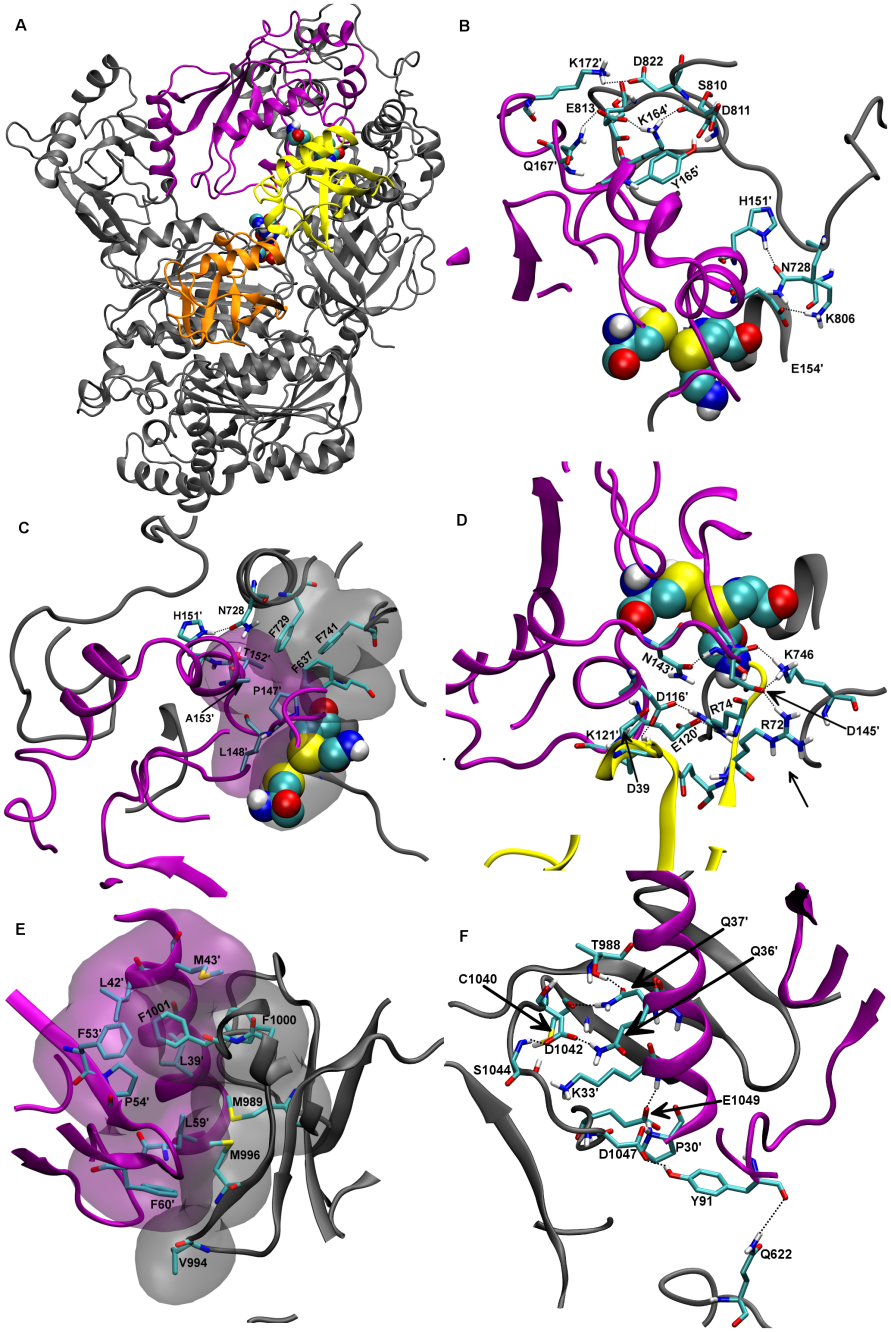
Final refined model of the tetrameric complex. A) Average structure of the last 20 ns of MD simulation of the model after SMD. B) Detail of the UbcH10-Cys-cap loop interactions. C) Detail of UbcH10-Cys region involved in hydrophobic interactions; D) Detail of the UbcH10-Cys region involved in polar interactions; E) Detail of the hydrophobic interactions between hUbA1 UFD and UbcH10. F) Detail of the polar interactions between hUbA1 UFD and UbcH10. Colour code: hUbA1, grey; Ub(T) yellow; Ub(A), orange; UbcH10, violet. Catalytic cysteins were highlighted in spheres. Apolar hydrogens were omitted for the sake of clarity. The van der Waals interactions are highlighted with transparent Connolly surfaces.

The first interface (Figure 6) comprises the UbcH10 residues Lys33′ and Gln37′, which are experimentally known to be critical for the interaction between E1 and E2 [41]–[43]: Lys33′ interacts with Asp1042 and with Ser1044, and Gln37′ is hydrogen-bonded to the backbone oxygen of Cys1040 and the hydroxyl group of Thr988 (Figure 6-F). Moreover, hydrogen bonds were also formed between the side chains of Gln36' and Asp1042, between Tyr91 and Asp1047, and between the N-terminal Pro30′ with Glu1049 (Figure 6-F). Finally, the ionic interactions were supplemented by hydrophobic contacts involving hUbA1 residues Met989, Val994, Met996, Phe1000, Phe1001, and UbcH10 residues Leu42′, Pro54′, Leu59′ and Phe60′ (Figure 6-E).

Interactions between the hUbA1 SCCH domain and the region surrounding the E2 catalytic cysteine were mainly characterized by a number of ionic and polar interactions between residues from H3 and H4 helices of UbcH10 (Glu154', Lys164', Lys172' and Tyr165') and residues from the hUbA1 Cys-cap loop (Gln728, Lys806, Glu813, Asp811 and Asp822) (Figure 6-B), while the region around the UbcH10 catalytic cysteine, including residues in the 3–10 helix, were involved in interactions with the residues around hUbA1 Cys632 and Ub(T). In particular, two main clusters of interactions are formed: i) the first, mainly based on hydrophobic interactions, between the UbcH10 helix H3 (Pro147′, Ala153′ and T150′) with the UbA1 coiled stretch between H24 and H25 (Phe637, Phe729 and Phe741), also supported by hydrogen bonds between Asn728 with His151′ and Thr150′ (Figure 6-C); ii) the second mainly involves residues closer to the catalytic cysteines, such as the ionic contact between the UbcH10 Asp145′ with UbA1-Lys746 and Ub(T)-Arg 72, and interactions between charged residues in the UbcH10 3–10 helix (Asp116′, Asp120′ and Lys121′) with Ub(T) residues (Gln40, Arg74, Asp39) and with the hUbA1 FCCH domain (Glu237) (Figure 6-D).

**Figure 7:**
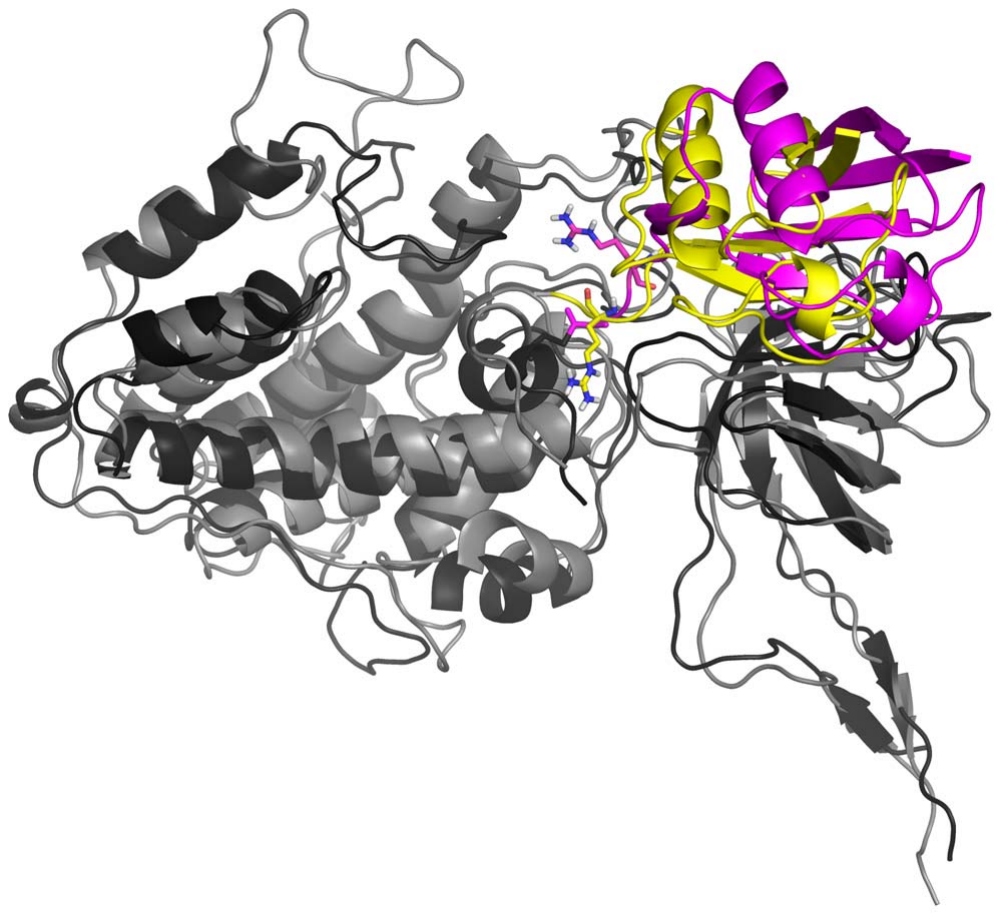
Rotation of the Ub(T) induced by UbcH10 interaction in the final quaternary model with respect to the UbA1∼Ub(T) model. Superimposition was made on the Cα atoms of the SCCH and FCCH domains. Colour code: Final quaternary complex model: hUbA1, grey; UbA1∼Ub(T) model: UbA1, black; Ub(T), magenta; Arg74 in the two models is shown as licorice. Apolar hydrogens were omitted for sake of clarity.

These findings demonstrated that the crosslinked Ub plays a key role in the transthiolation intermediate with UbcH10. In particular, MD simulations highlighted that the approach of the catalytic cysteines induced a rotation of 25° of Ub(T) with respect to hUbA1 and a rearrangement of the Ub(T) pattern of interactions showed in models lacking E2 (Figure 7). In particular, in absence of E2 Arg74 was hydrogen-bonded to Cys-cap residues (Asp811 and Gln812), while in the final model it was involved in ionic interactions with UbcH10-Glu120′ and Asp116′, and with Glu237, bearing to the hUbA1 FCCH domain. These data support the hypothesis that products of the transthiolation reaction might be released upon a process involving the rearrangement of the Ub(T) binding to E1, driven by the charged residues in the region surrounding the catalytic cysteine of E2. Finally, we also observed some interactions in the loop region of hUbA1, in particular hydrogen bonds between the side chain of Asn622 with the backbone of UbcH10-Tyr 91′ (Figure 6-F), and between the backbone of Ser628 and the side chain of Glu120' (Figure 6-D). A representative snapshot of the 3D model is available as supplemental PDB file.

### Peptide affinity assays

On the basis of the 3D model of the quaternary complex, we have designed six peptides as molecular probes in order to calibrate their ability to interfere the binding of UbcH10. This strategy was motivated by two main reasons. First, the identification of short peptides that mediate protein-protein interaction seemed *a priori* effective for disrupting the protein-protein recognition and binding. While other strategies, i.e. introduction of specific mutations, may also be envisaged, it is unclear whether single-point mutants might lead to a significant destabilization of the complex or even to impede the formation of the quaternary complex. Second, since our ultimate goal is the design of compounds that might disrupt the ubiquitilation process, testing a series of suitably chosen short peptides represents a valuable proof-of-concept for supporting the potential therapeutic effect of peptidomimetics. Specifically, the peptides were designed to examine the capability of hUbA1 stretches that contribute to the protein-protein interface with UbcH10 (Figure S7). In particular, we have designed two peptides per interface, which will be denoted S for SCCH region, L for cross loop, and U for UFD (Table 4). In the UFD region peptides U1 and U2 were selected to test the relevance of the acidic residues in mediating the binding of the UbcH10 H1 helix. In the SCCH region peptide S1 was chosen to test the role of the Cys-cap in binding UbcH10, while peptide S2, corresponding to helix H19 in the SCCH region, was designed as negative control, since the 3D model revealed the lack of any interaction in the complex. Finally, peptides L1 and L2 were chosen to explore the role of the cross loop region in assisting the interaction with UbcH10. All the peptides were synthesized as biotinylated derivatives by solid phase method and purified by RP-HPLC. Unfortunately, S1 and U2 were insoluble and so not testable in binding assays. The ability of the soluble peptides to bind recombinant GST-UbcH10 was checked by ELISA, utilizing GST as control (data not shown).

**Table 4:** Sequence and binding assays results of the designed peptides.

UbA1 region	peptide	sequence	K_D_ (µM)
UFD	U1	1038-LCCNDESGEDVEV-1050	10
	U2	1036-LELCCNDESGEDV-1048	Not soluble
SCCH	S1	807-IHVSDQELQSA-817	Not soluble
	S2	649-RDEFEGLFKQPAEN-662	No binding
LOOP	L1	621-SQDPPEKSIPI-631	No binding
	L2	615-TESYSSSQDPPEK-627	20
Scrambles	ScrU1	ELNCDVEVEGSDC	>100
	ScrL2	SDPSKTSEYQPSE	>80

Numbering is referred to the human UbA1 sequence. K_D_ values are calculated by Elisa assays.

The best results (Table 4) were obtained with U1 and L2, which were found to bind UbcH10 with an apparent K_D_ of about 10 and 20 µM, respectively. In order to confirm that the binding of U1 and L2 peptides was sequence-dependent, two scrambled peptides were synthesized, ScrU1 and ScrL2. The results demonstrated that these peptides exhibited a very poor binding, much weaker than U1 and L2, which might then be considered indicative of native protein-protein interactions. In particular the good affinity showed by U1 allowed us to validate the role of the acidic residues of the UFD region in binding E2, thus giving confidence to our 3D model. The U1 peptide, indeed, contained D1047 and E1049, two of the three acidic residues involved in the hUbA1 UFD-UbcH10 H1 interface. Unfortunately, the low solubility of U2 did not allow us to verify the role of E1037, which is the third residue proven to be involved in the interaction by mutagenesis studies. Similarly, the results obtained for L2 support the role of Gln622 in assisting the interaction of the crossover loop with UbcH10, in agreement with the 3D model. The low affinity showed by L1 peptide, which contains Gln622 at the N-terminus side of the sequence, might be indicative of the importance of flanking residues in L2 binding. Finally, the results obtained from the SCCH peptides allowed us to exclude a role in the binding of the helix region corresponding to S2, as expected for this peptide, which was designed as negative control.

Overall, the results support the involvement of the selected peptides in mediating the protein-protein interactions in the hUbA1∼Ub(T)-Ub(A)-UbcH10, which in turn reinforces the reliability of the 3D model built up for the quaternary complex between E1, E2 and Ub partners. On the other hand, they also demonstrate the feasibility of interfering the formation of the complex, which paves the way to the structure-based design of peptidomimetics for UbcH10-related anticancer strategies.

## Conclusions

We have simulated the dynamic process associated to the formation of the complex leading to the transthiolation reaction between doubly loaded hUbA1 and UbcH10. The formation of the complex takes place through protein-protein interactions in three main interfaces: i) the first between the hUbA1 UFD domain and the UbcH10 helix H1 and β1β2 loop, ii) the second formed by the hUbA1 SCCH domain and Ub(T) with the region surrounding the UbcH10 Cys114', involving residues from the 3–10 helix and helices H2 and H3, and iii) the third between the hUbA1 crossing loop and Ub(A) with UbcH10. The involvement of these regions has been supported by the ELISA assays performed for a series of short peptides that encompass the residues that mediate the interaction between UbA1, UbcH10 and the two Ubs. In particular, peptides U1 and L2, pertaining to the UBA1 UFD domain and to the UbA1 loop, have been able to interfere the assembly of the E1–E2 complex. The availability of this structural model should facilitate the understanding of the structural details of the ubiquitination cascade, to rationalize the details of the recognition between E1 and E2 partners, and finally to facilitate the design of peptidomimetics or small size compounds able to interfere with the formation of the E1–E2 complex, which might be valuable to open new strategies against tumorigenic processes.

## Supporting Information

Figure S1 **Comparison of the Ramachandran plot of the models A (A) and C (C) with the corresponding conformations of the template 3CMM (B and D respectively).**
(TIFF)

Figure S2 A: Time evolution (ns) of the RMSD (Å) of the UbA1_C model: hUbA1 apo (red), hUbA1∼Ub(T) (yellow), hUbA1∼Ub(T)-Ub(A) (green) and hUbA1∼Ub(T)-Ub(A)-UbcH10 (black). B, C and D: Structural preservation of the structure of each region. of: hUbA1 apo (B), hUbA1-Ub(T) (C) and UbA1∼Ub(T)-Ub(A) (D), AD (black), UFD (green), SCCH (red) and FCCH (blue).
(TIFF)

Figure S3 **Superposition of the backbone for the X-ray structure 4NNJ and the 3D model of the ternary complex.** Front and side views are shown in the left and right pictures, respectively. Colour code: hUbA1, grey; Ub(T) yellow; Ub(A), orange; scUbA1, magenta scUb(A), green, scUb(A), Cyan.
(TIFF)

Figure S4 **Analysis of the distance between the sulphur atom of the UbcH10 Cys114 and the carbonyl group of the crosslinked Ub(T) terminal glycine during the 500 ns unconstrained MD.**
(TIFF)

Figure S5 **Analysis of the distance between the sulphur of the UbcH10 Cys114 and the carbonyl group of the crosslinked Ub(T) C-terminal glycine during the 50 ns unconstrained MD of the final model obtained after SMD.**
(TIFF)

Figure S6 **Superposition of the UbA1 and Ub(A) backbone for the X-ray structure 4II2 (grey) and the 3D model of the quaternary complex (Green).** Front and rear views are shown in the left and right pictures, respectively.
(TIFF)

Figure S7 **Strategy of peptide design, highlights of the hUbA1 regions used to design the peptides.** Colour code: hUbA1, grey; Ub(T) yellow; Ub(A), orange; UbcH10, violet; S1 and S2 red; U1 and U2 green; L1 and L2 blue.
(TIF)

Table S1 **List of the active residues used in each docking step.**
(DOCX)

Table S2 **Results from HADDOCK calculations performed for the dimeric hUbA1 and Ub(T) complex.** Clusters of poses are given ordered by total score. The best models are highlighted in bold.
(DOCX)

Table S3 **Time evolution of interaction surface (Å^2^) for selected domains in hUbA1.**
(DOCX)

File S1 **Atomic Coordinates of a representative snapshot of the 3D model.**
(PDB)
