# Supplementary figures and images for: Structural Model of the hUbA1-UbcH10 Quaternary Complex: In Silico and Experimental Analysis of the Protein-Protein Interactions between E1, E2 and Ubiquitin
Source: PLoS One. 2014 Nov 6;9(11):e112082. doi: 10.1371/journal.pone.0112082 (PMC4223017; doi:10.1371/journal.pone.0112082)

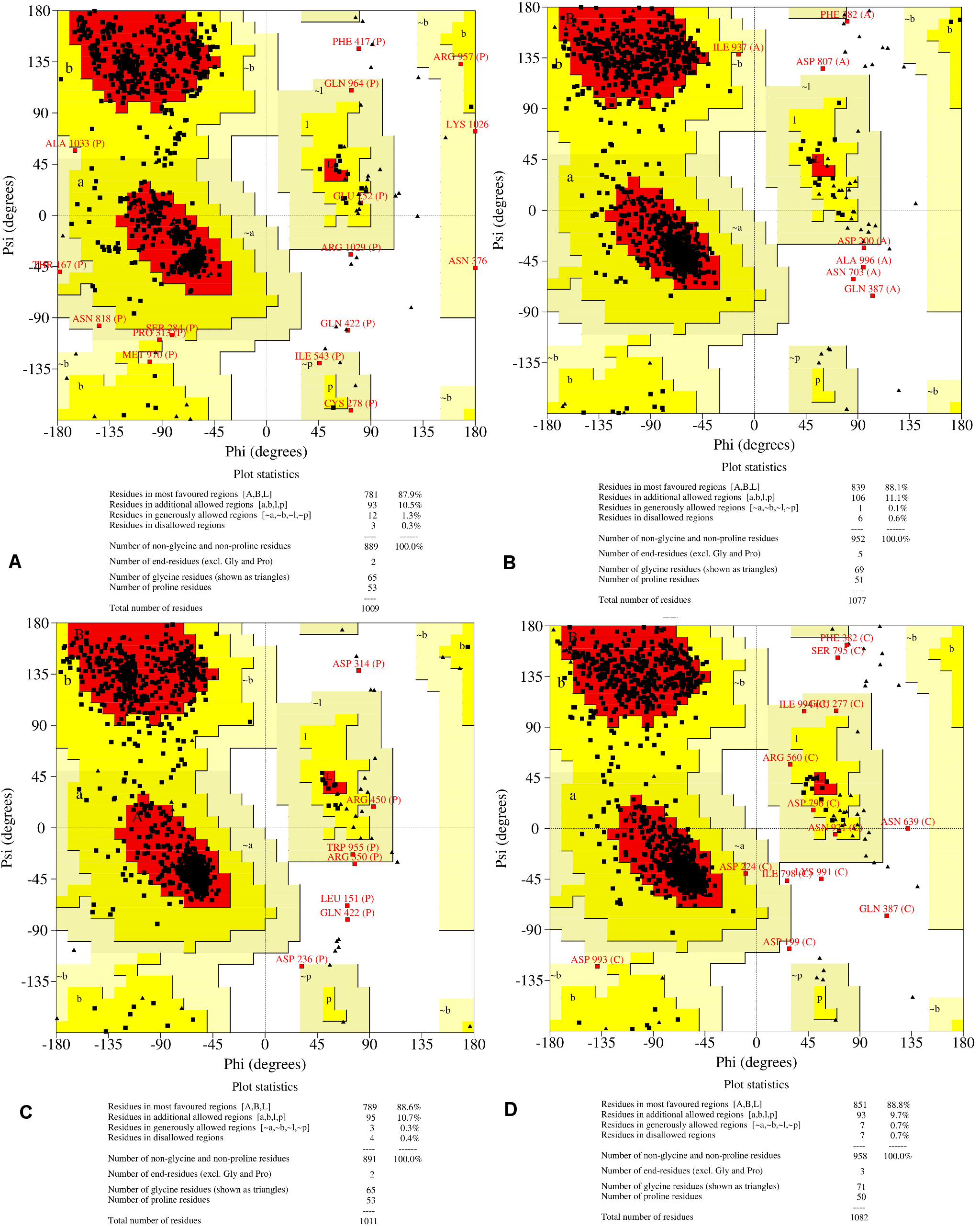

Supplement: Figure S1 — Comparison of the Ramachandran plot of the models A (A) and C (C) with the corresponding conformations of the template 3CMM (B and D respectively). (TIFF) [file pone.0112082.s001.tiff]

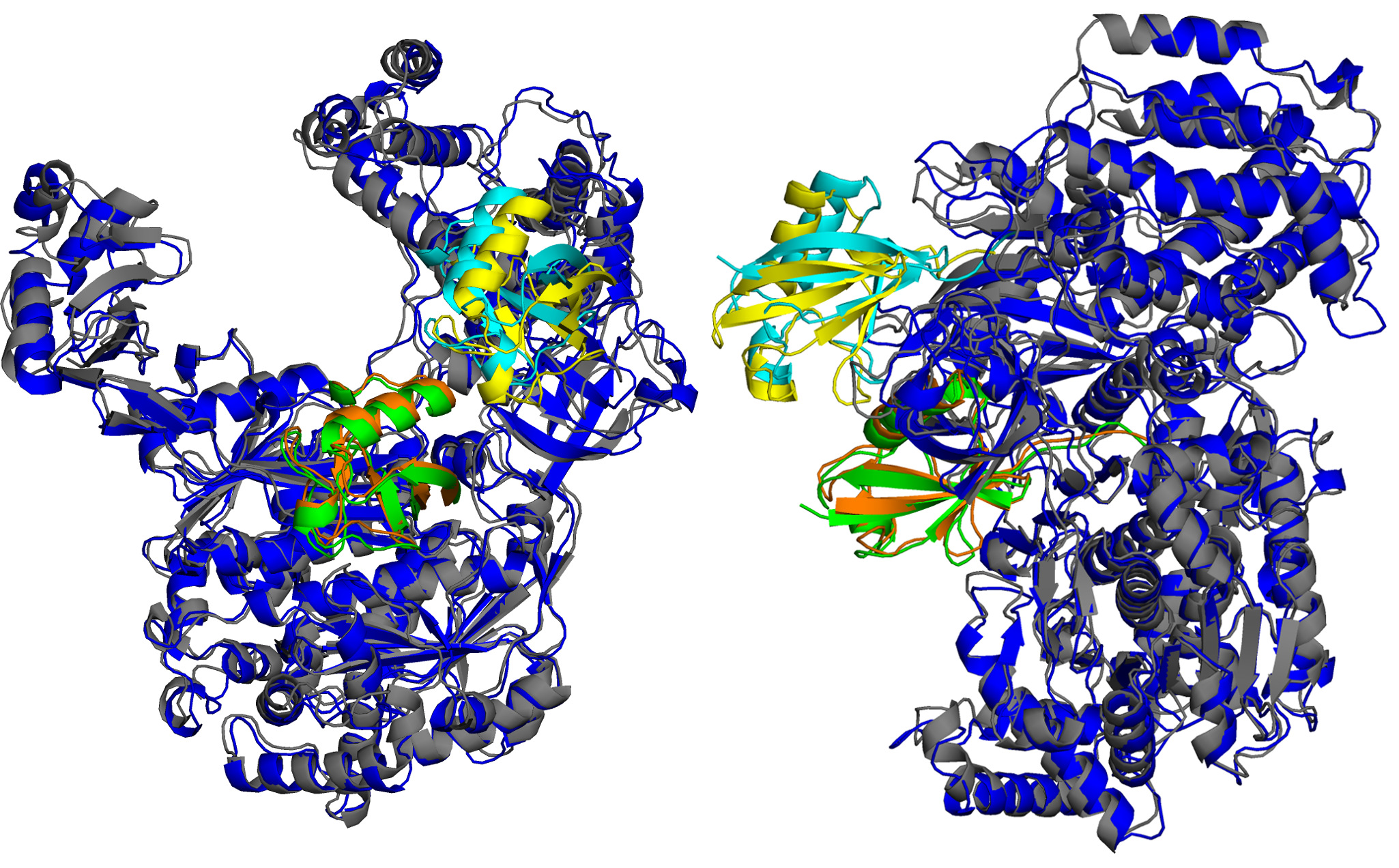

Supplement: Figure S3 — Superposition of the backbone for the X-ray structure 4NNJ and the 3D model of the ternary complex. Front and side views are shown in the left and right pictures, respectively. Colour code: hUbA1, grey; Ub(T) yellow; Ub(A), orange; scUbA1, magenta scUb(A), green, scUb(A), Cyan. (TIFF) [file pone.0112082.s003.tiff]

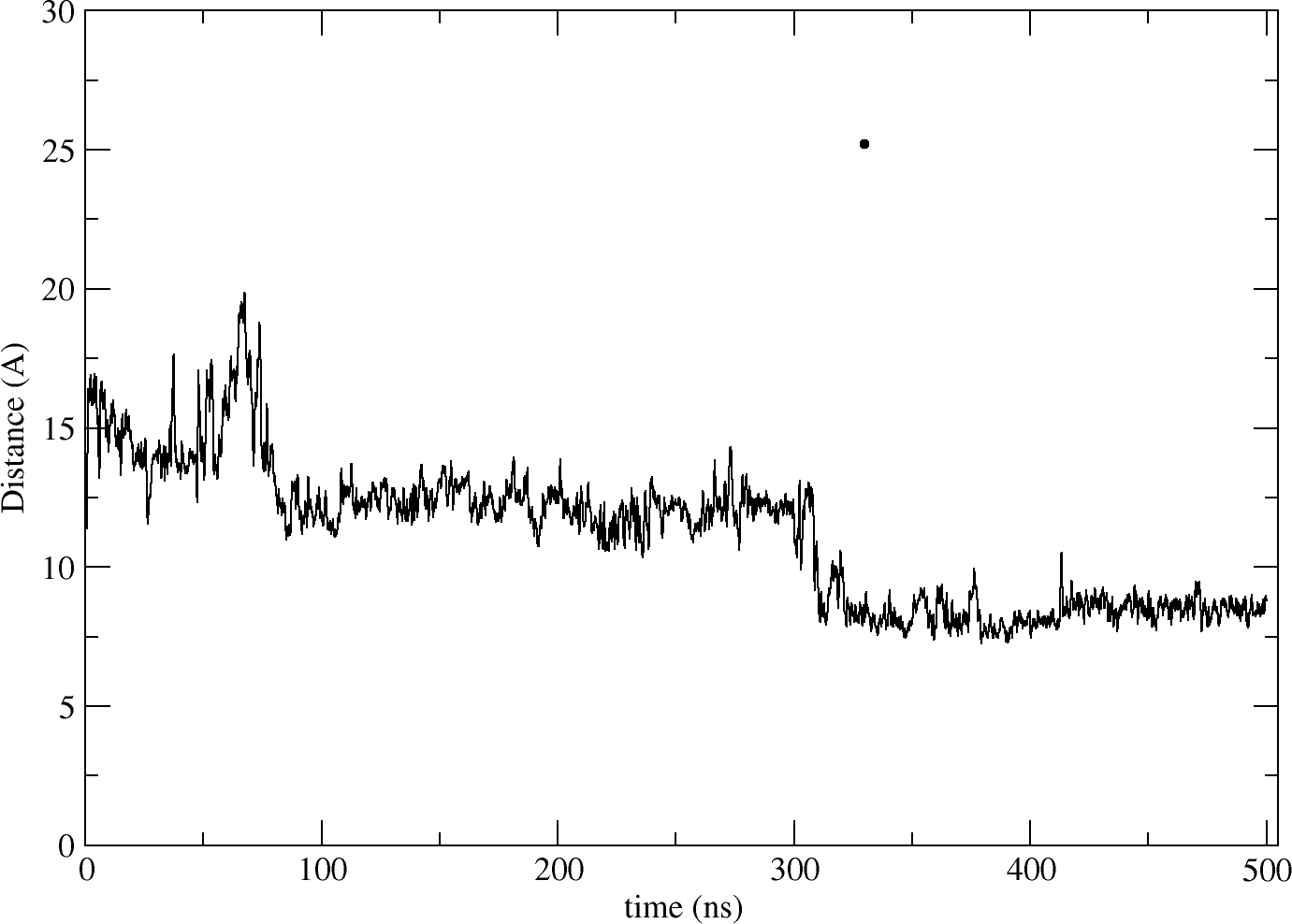

Supplement: Figure S4 — Analysis of the distance between the sulphur atom of the UbcH10 Cys114 and the carbonyl group of the crosslinked Ub(T) terminal glycine during the 500 ns unconstrained MD. (TIFF) [file pone.0112082.s004.tiff]

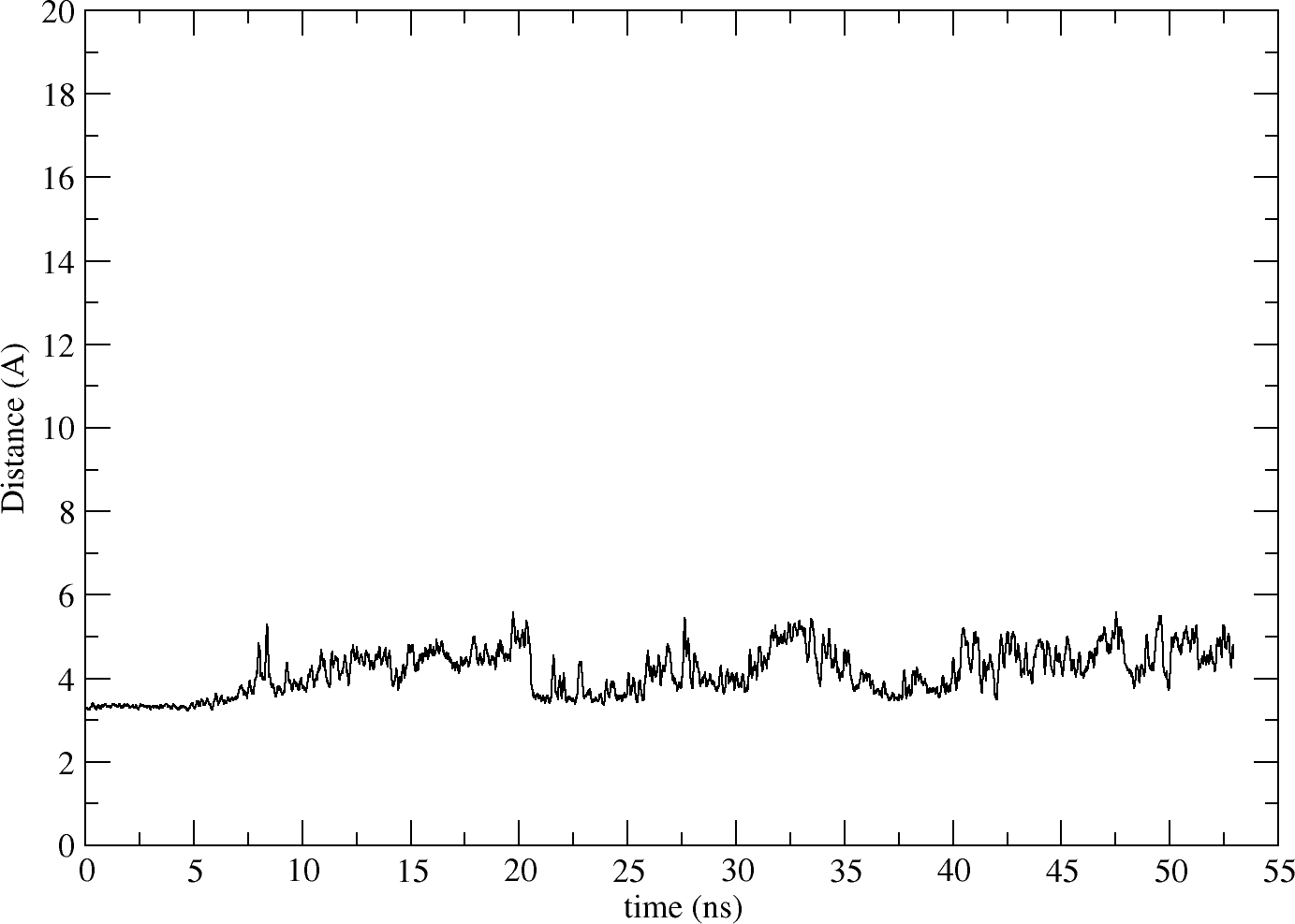

Supplement: Figure S5 — Analysis of the distance between the sulphur of the UbcH10 Cys114 and the carbonyl group of the crosslinked Ub(T) C-terminal glycine during the 50 ns unconstrained MD of the final model obtained after SMD. (TIFF) [file pone.0112082.s005.tiff]

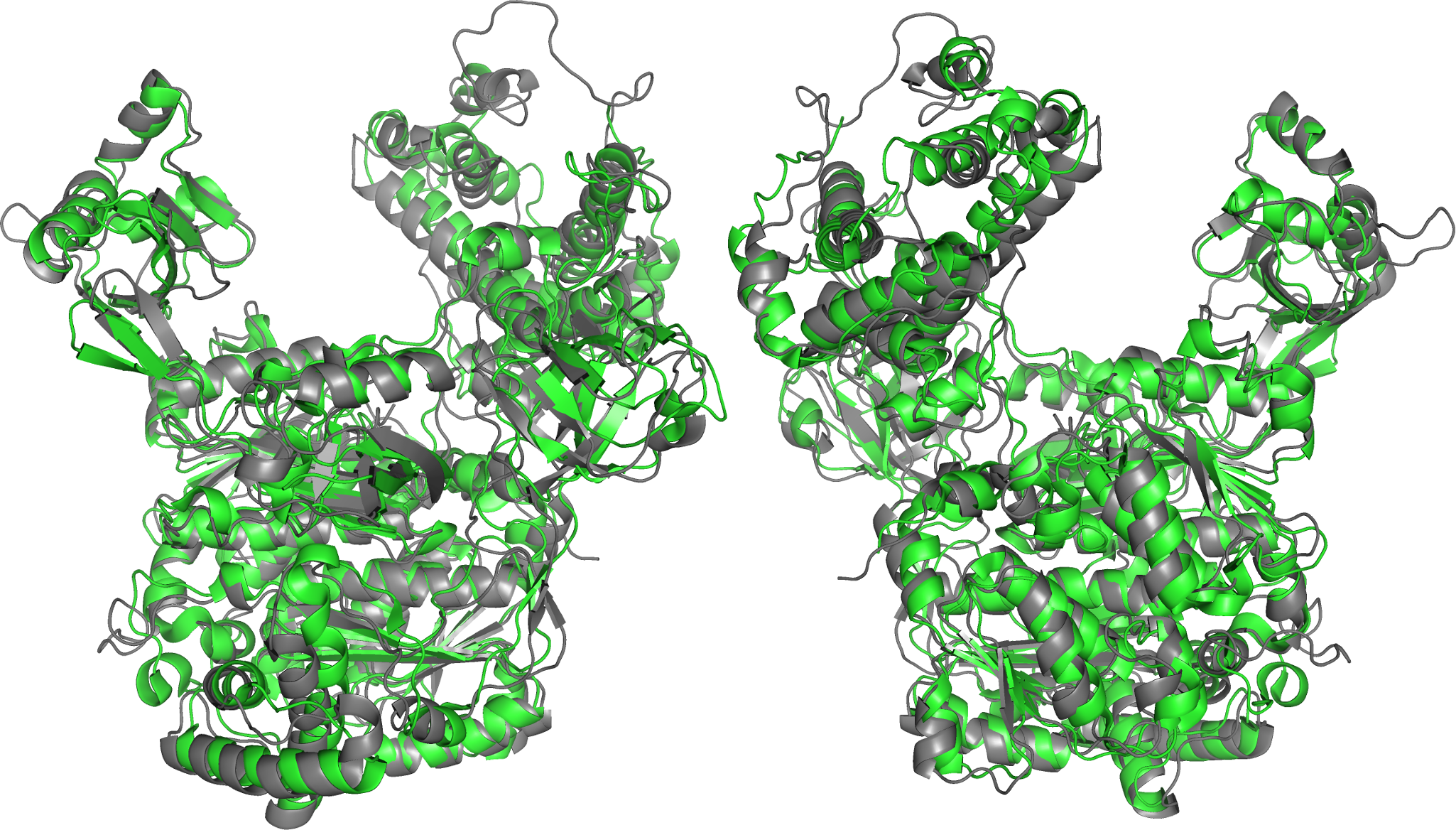

Supplement: Figure S6 — Superposition of the UbA1 and Ub(A) backbone for the X-ray structure 4II2 (grey) and the 3D model of the quaternary complex (Green). Front and rear views are shown in the left and right pictures, respectively. (TIFF) [file pone.0112082.s006.tiff]

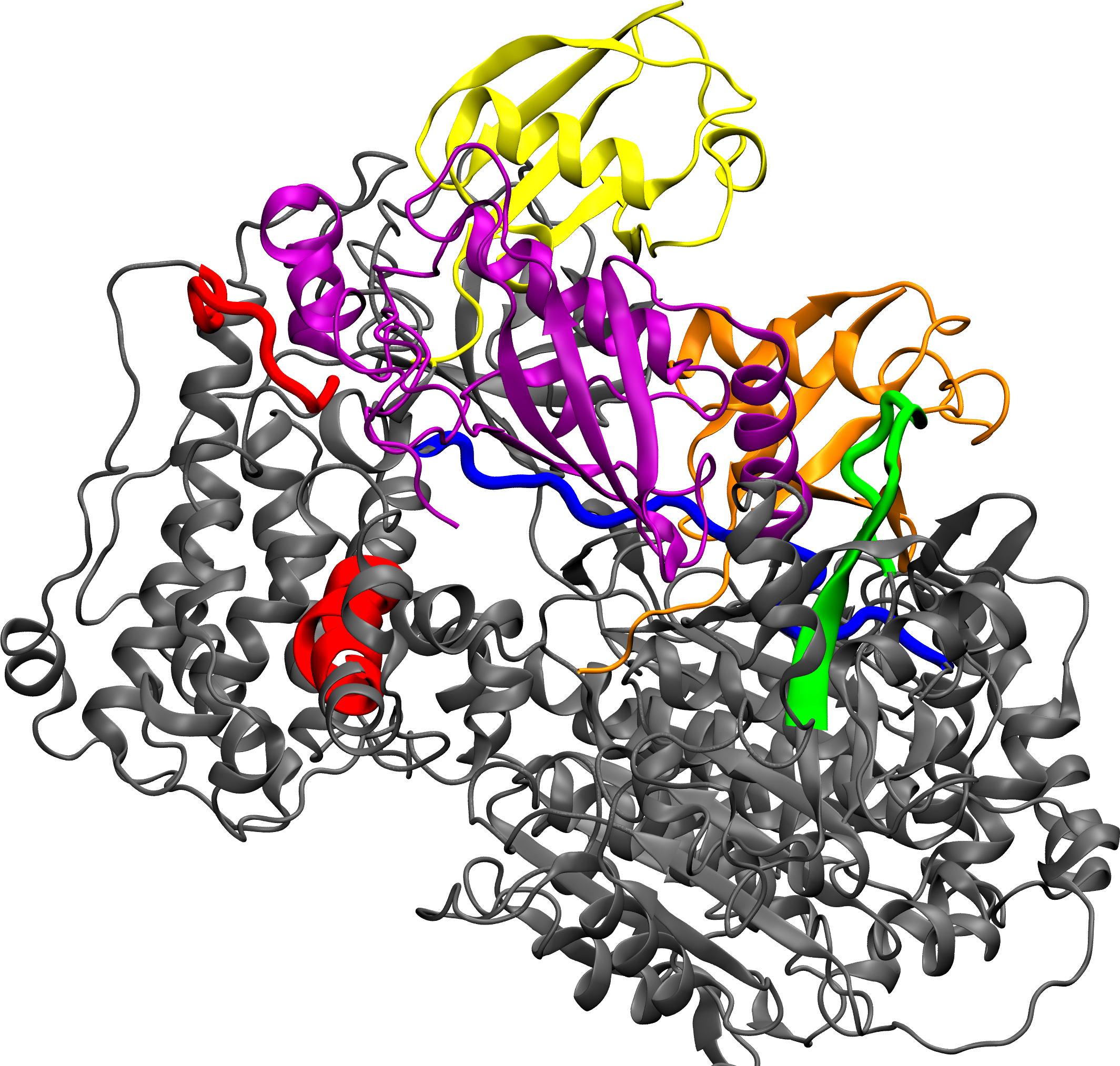

Supplement: Figure S7 — Strategy of peptide design, highlights of the hUbA1 regions used to design the peptides. Colour code: hUbA1, grey; Ub(T) yellow; Ub(A), orange; UbcH10, violet; S1 and S2 red; U1 and U2 green; L1 and L2 blue. (TIF) [file pone.0112082.s007.tif]
