# Supplementary material for: Structural Model of the hUbA1-UbcH10 Quaternary Complex: In Silico and Experimental Analysis of the Protein-Protein Interactions between E1, E2 and Ubiquitin
Source: PLoS One. 2014 Nov 6;9(11):e112082. doi: 10.1371/journal.pone.0112082 (PMC4223017; doi:10.1371/journal.pone.0112082)
Supplement: Table S1 — List of the active residues used in each docking step. (DOCX) [file pone.0112082.s008.docx]

**Table S1** List of the active residues used in each docking step.

| Docking step | UbA1 | Ub(T) | Ub(A) | UbcH10 |
| --- | --- | --- | --- | --- |
| dimeric complex  UbA1~Ub(T) | Cys632 | Gly76 |  |  |
| trimeric complex  UbA1~Ub(T)-Ub(A) | Arg239 |  | Asp32 |  |
|  | Asp576 |  | Arg72 |  |
|  | Tyr600 |  | Gly75 |  |
|  |  |  | Gly76 |  |
| tetrameric complex  UbA1~Ub(T)-Ub(A)-UbcH10 | Glu1037 |  |  | Lys33’ |
|  | Asp1047 |  |  | Gln37’ |
|  | Glu1049 |  |  |  |
|  | Cys632 |  |  | Cys114’ |
