# Supplementary material for: Structural Model of the hUbA1-UbcH10 Quaternary Complex: In Silico and Experimental Analysis of the Protein-Protein Interactions between E1, E2 and Ubiquitin
Source: PLoS One. 2014 Nov 6;9(11):e112082. doi: 10.1371/journal.pone.0112082 (PMC4223017; doi:10.1371/journal.pone.0112082)
Supplement: Table S2 — Results from HADDOCK calculations performed for the dimeric hUbA1 and Ub(T) complex. Clusters of poses are given ordered by total score. The best models are highlighted in bold. (DOCX) [file pone.0112082.s009.docx]

**Table S2**. **HADDOCK results for the dimeric complex hUbA1-Ub(T)**. The structural data include the distance (Å) from the sulphur of the hUbA1 Cys632 to the C-terminal Gly76 of Ub(T). The energy data report the score of the docked structures obtained from HADDOCK and from SIE (kcal/mol). Lowest energy result for clusters 3 and 2 (named respectively Ha and Hb) were selected for Rosetta calculations.

| **HADDOCK Cluster** | **population** | **UbcH10 S-C114 Ub(T) C-ter G76** | **Haddock Score** | **SIE**  **ΔG** |
| --- | --- | --- | --- | --- |
| 3 | 32 | 3,7 | -47,6 +/- 4,6 | -4,88 |
| 5 | 5 | 4,2 | -32,6 +/- 6,0 | 1,78 |
| 2 | 62 | 3,6 | -31,9 +/- 1,2 | -6,71 |
| 6 | 5 | 4,3 | -26 +/- 6,5 | -4,07 |
| 8 | 4 | 4,1 | -25,8 +/- 2,4 | -5,57 |
| 4 | 10 | 4,7 | -19,8 +/- 6,3 | -6,91 |
| 1 | 73 | 4,7 | -17,7 +/- 3,6 | -4,89 |
| 7 | 5 | 6,4 | -2,3 +/- 5,3 | -5,1 |
