# Supplementary material for: Structural Model of the hUbA1-UbcH10 Quaternary Complex: In Silico and Experimental Analysis of the Protein-Protein Interactions between E1, E2 and Ubiquitin
Source: PLoS One. 2014 Nov 6;9(11):e112082. doi: 10.1371/journal.pone.0112082 (PMC4223017; doi:10.1371/journal.pone.0112082)
Supplement: Table S3 — Time evolution of interaction surface (Å2) for selected domains in hUbA1. (DOCX) [file pone.0112082.s010.docx]

**Table S3** Time evolution of interaction surface (Å^2^) for selected domains in hUbA1.

| time (ns) | **TOTAL interface** | **UFD interface** | **SCCH interface** | % UFD | % SCCH |
| --- | --- | --- | --- | --- | --- |
| docking | **2518** | **1301** | **703** | 52 | 28 |
| 100 | **2662** | **1391** | **588** | 52 | 22 |
| 200 | **3211** | **1628** | **766** | 51 | 24 |
| 300 | **3278** | **1625** | **957** | 50 | 29 |
| 400 | **2964** | **1557** | **728** | 53 | 25 |
| 500 | **2659** | **1571** | **724** | 59 | 27 |
| Final Complex | **5437** | **1558** | **3162** | 29 | 58 |
| X-ray (4II2) | **3326** | **1154** | **1715** | 35 | 52 |
